# Supplementary material for: Habitat suitability—density relationship in an endangered woodland species: the case of the Blue Chaffinch (Fringilla polatzeki)
Source: PeerJ. 2017 Sep 12;5:e3771. doi: 10.7717/peerj.3771 (PMC5600174; doi:10.7717/peerj.3771)

**Supplementary material**.

**Table S1.** Environmental characteristics of the four studied pine forests (Inagua, 50*50 m cells = 15,037; Tamadaba, 11,246; Pilancones, 12,667; Tauro, 1,880), the areas traversed by the survey trail in Inagua reserve (n = 100), and of the nests with successful breeding attempts in Inagua (n = 59).

|  |  | Inagua | Tamadaba | Pilancones | Tauro | survey trail | nests |
| --- | --- | --- | --- | --- | --- | --- | --- |
| Altitude | mean | 1115.50 | 1013.38 | 1008.15 | 911.99 | 1259.00 | 1264.92 |
| (m) | min | 250.00 | 360.00 | 300.00 | 295.00 | 1075.00 | 865.00 |
|  | max | 1550.00 | 1435.00 | 1510.00 | 1215.00 | 1450.00 | 1485.00 |
|  | sd | 168.13 | 198.93 | 178.15 | 167.79 | 95.07 | 142.43 |
| Slope | mean | 45.83 | 48.08 | 44.54 | 54.05 | 58.05 | 50.39 |
| (%) | min | 0.00 | 0.00 | 0.00 | 0.00 | 17.78 | 22.44 |
|  | max | 183.75 | 260.19 | 155.72 | 219.95 | 120.20 | 118.25 |
|  | sd | 23.44 | 24.95 | 22.78 | 32.90 | 23.10 | 15.96 |
| Western orientation | mean | -0.09 | 0.16 | -0.05 | -0.08 | -0.07 | 0.01 |
| (sin cardinal orientation) | min | -1.00 | -1.00 | -1.00 | -1.00 | -0.98 | -0.97 |
|  | max | 1.00 | 1.00 | 1.00 | 1.00 | 0.98 | 0.95 |
|  | sd | 0.70 | 0.68 | 0.64 | 0.65 | 0.71 | 0.73 |
| Northern orientation | mean | 0.03 | -0.06 | -0.03 | 0.02 | -0.03 | -0.16 |
| (cos cardinal orientation) | min | -1.00 | -1.00 | -1.00 | -1.00 | -0.98 | -0.94 |
|  | max | 1.00 | 1.00 | 1.00 | 1.00 | 0.99 | 0.97 |
|  | sd | 0.70 | 0.71 | 0.77 | 0.76 | 0.58 | 0.57 |
|  |  |  |  |  |  |  |  |
| Cover of the canopy (pine) layer | mean | 25.79 | 42.74 | 18.38 | 16.78 | 29.56 | 34.43 |
| (%) | min | 0.00 | 0.27 | 0.41 | 0.39 | 2.91 | 17.65 |
|  | max | 97.26 | 99.29 | 82.00 | 80.49 | 46.69 | 52.64 |
|  | sd | 13.87 | 20.10 | 10.33 | 11.32 | 8.46 | 5.86 |
| Average pine height | mean | 16.50 | 14.26 | 13.57 | 13.49 | 17.84 | 21.04 |
| (m) | min | 0.00 | 2.16 | 2.00 | 2.30 | 6.50 | 14.32 |
|  | max | 40.23 | 36.66 | 31.64 | 29.96 | 26.64 | 25.95 |
|  | sd | 6.38 | 4.66 | 4.15 | 4.88 | 4.96 | 2.41 |
| Cover of the shrub layer | mean | 8.83 | 13.42 | 6.05 | 8.67 | 8.47 | 9.81 |
| (%) | min | 0.00 | 0.00 | 0.00 | 0.00 | 0.67 | 0.71 |
|  | max | 75.00 | 64.00 | 59.00 | 63.00 | 22.55 | 44.17 |
|  | sd | 9.98 | 10.70 | 7.63 | 9.63 | 6.16 | 8.43 |
| Average height of shrubs | mean | 0.70 | 0.74 | 0.68 | 0.71 | 0.70 | 0.71 |
| (m) | min | 0.00 | 0.57 | 0.53 | 0.49 | 0.62 | 0.62 |
|  | max | 1.25 | 1.22 | 1.21 | 1.25 | 0.83 | 1.01 |
|  | sd | 0.09 | 0.10 | 0.07 | 0.09 | 0.06 | 0.07 |
| Incident solar radiation | mean | 7008.74 | 6796.56 | 6788.95 | 6972.34 | 7030.16 | 7062.41 |
| (average April-August; kWh/m^2^) | min | 5230.27 | 4567.25 | 5260.99 | 5282.73 | 6197.41 | 6180.29 |
|  | max | 7435.98 | 7221.65 | 7208.58 | 7514.96 | 7317.35 | 7268.68 |
|  | sd | 264.54 | 280.17 | 252.71 | 400.39 | 238.69 | 171.37 |
|  |  |  |  |  |  |  |  |
| Average temperature in May | mean | 19.42 | 18.54 | 19.35 | 20.28 | 19.62 | 19.44 |
| (ºC) | min | 17.89 | 16.98 | 17.82 | 18.80 | 18.53 | 17.94 |
|  | max | 20.37 | 19.92 | 20.70 | 21.23 | 20.19 | 20.16 |
|  | sd | 0.67 | 0.68 | 0.66 | 0.41 | 0.46 | 0.59 |
| Average temperature in July | mean | 24.48 | 23.85 | 24.98 | 24.55 | 24.47 | 24.44 |
| (ºC) | min | 23.95 | 23.61 | 24.44 | 24.25 | 24.25 | 24.07 |
|  | max | 25.19 | 23.98 | 25.91 | 25.14 | 24.95 | 25.18 |
|  | sd | 0.26 | 0.08 | 0.35 | 0.19 | 0.19 | 0.20 |
| Rainfall in July-September | mean | 15.87 | 23.88 | 8.36 | 14.48 | 20.02 | 19.57 |
| (mm) | min | 3.00 | 11.00 | 0.00 | 1.00 | 13.18 | 8.07 |
|  | max | 28.00 | 34.00 | 17.00 | 20.00 | 27.00 | 26.85 |
|  | sd | 6.44 | 4.63 | 4.04 | 4.31 | 3.91 | 4.45 |

**Figures S1, S2 and S3**: Contour line maps representing the habitat suitability for the successful breeding of the blue chaffinch in four pine forests of Gran Canaria island. For the location of these study areas within Gran Canaria see Figure 1, and Table S1 for environmental characteristics. The suitability level of 0.5 denotes random distribution of the species. The contour lines have been applied to a map of 50*50 m cells. The suitability of each cell was obtained after smoothing the original prediction of the boosted classification trees (BCT), considering a larger square of 5*5 cells where the cell of interest was located in its center. BCT models were carried out with the habitat characteristics in pixels of 50*50 m around nests (59 nests with breeding success recorded in six years from 2011 to 2016) against the same number of pixels of the same size randomly obtained from the pine forests of Inagua reserve.


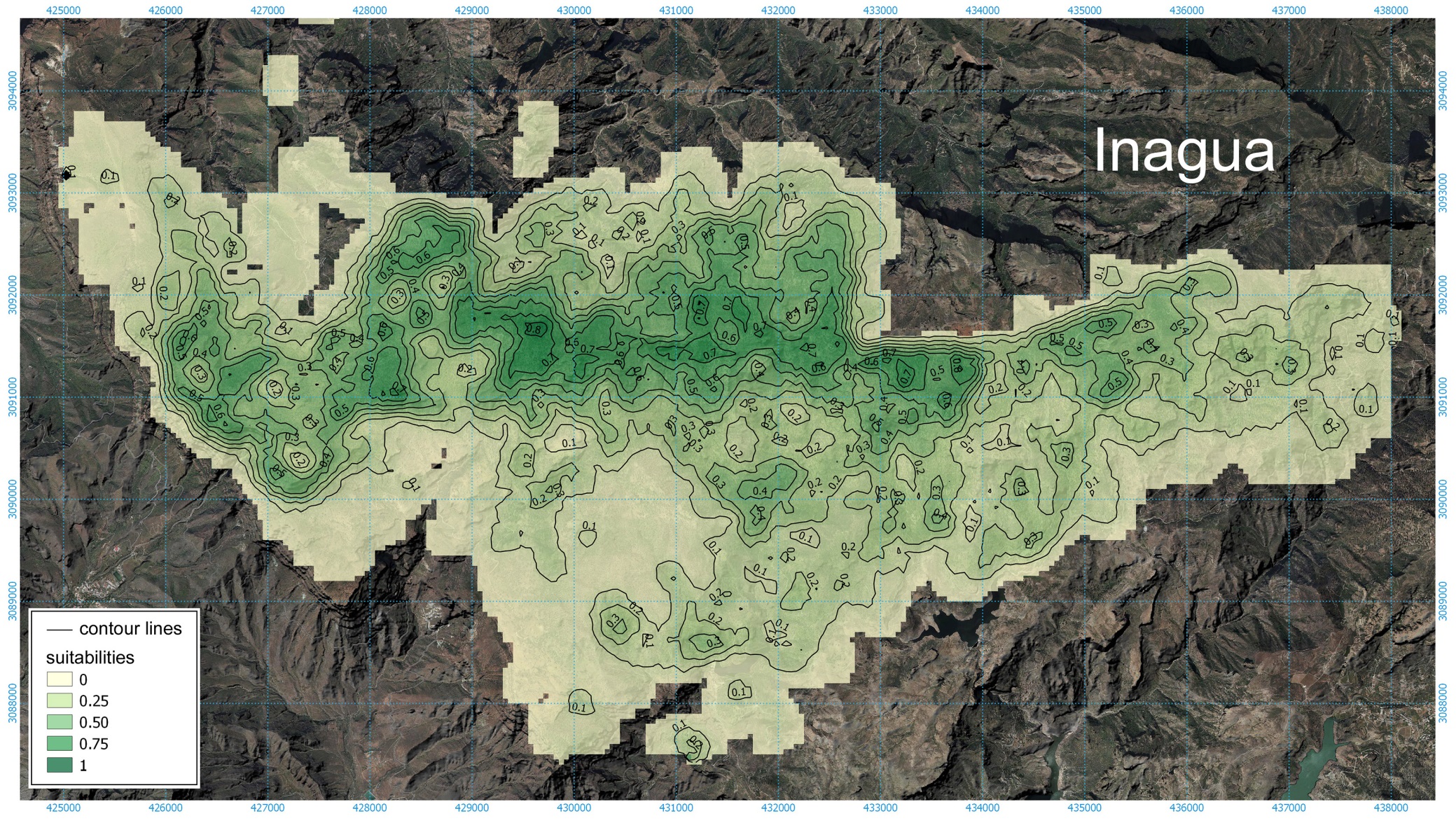


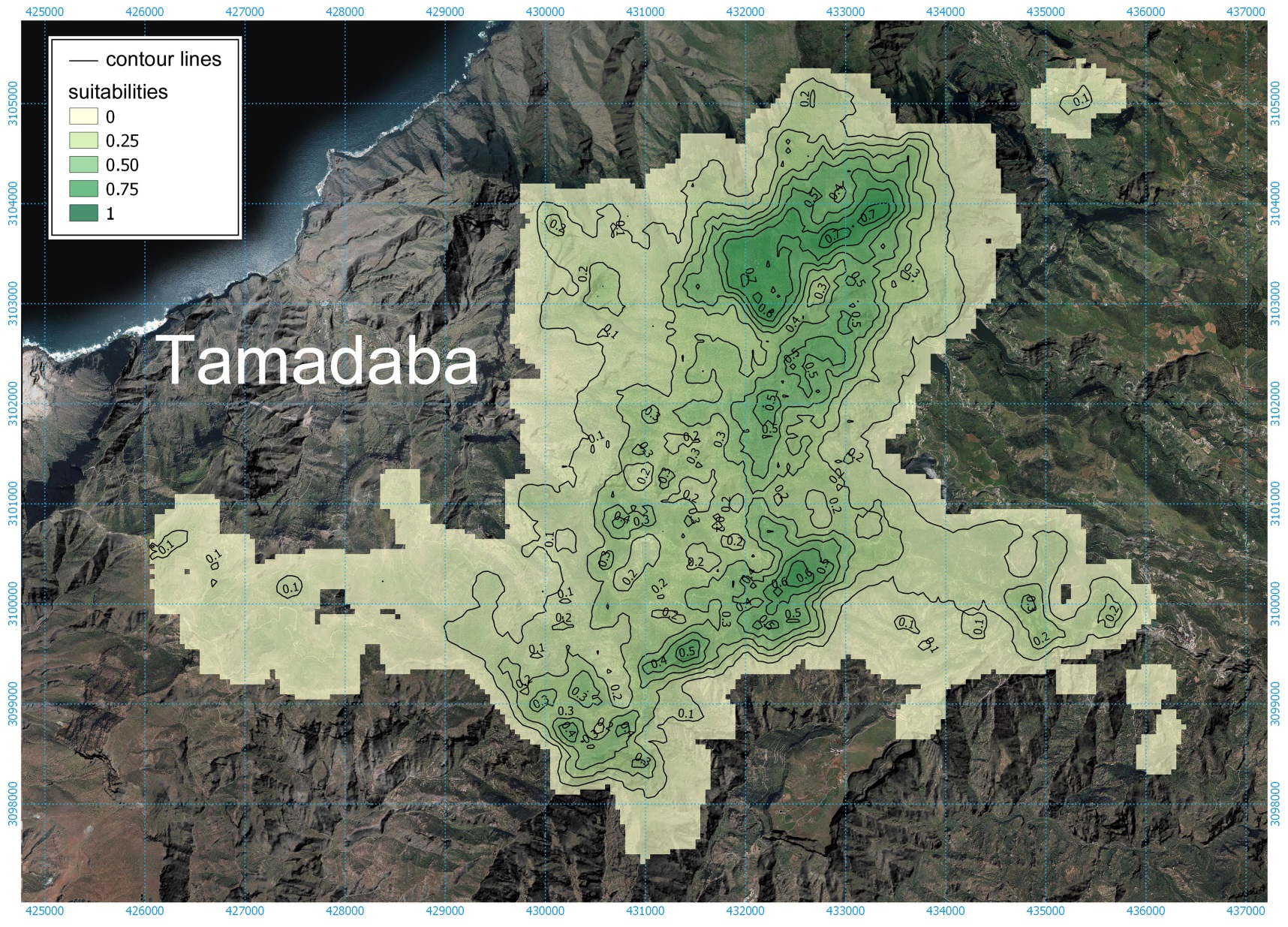


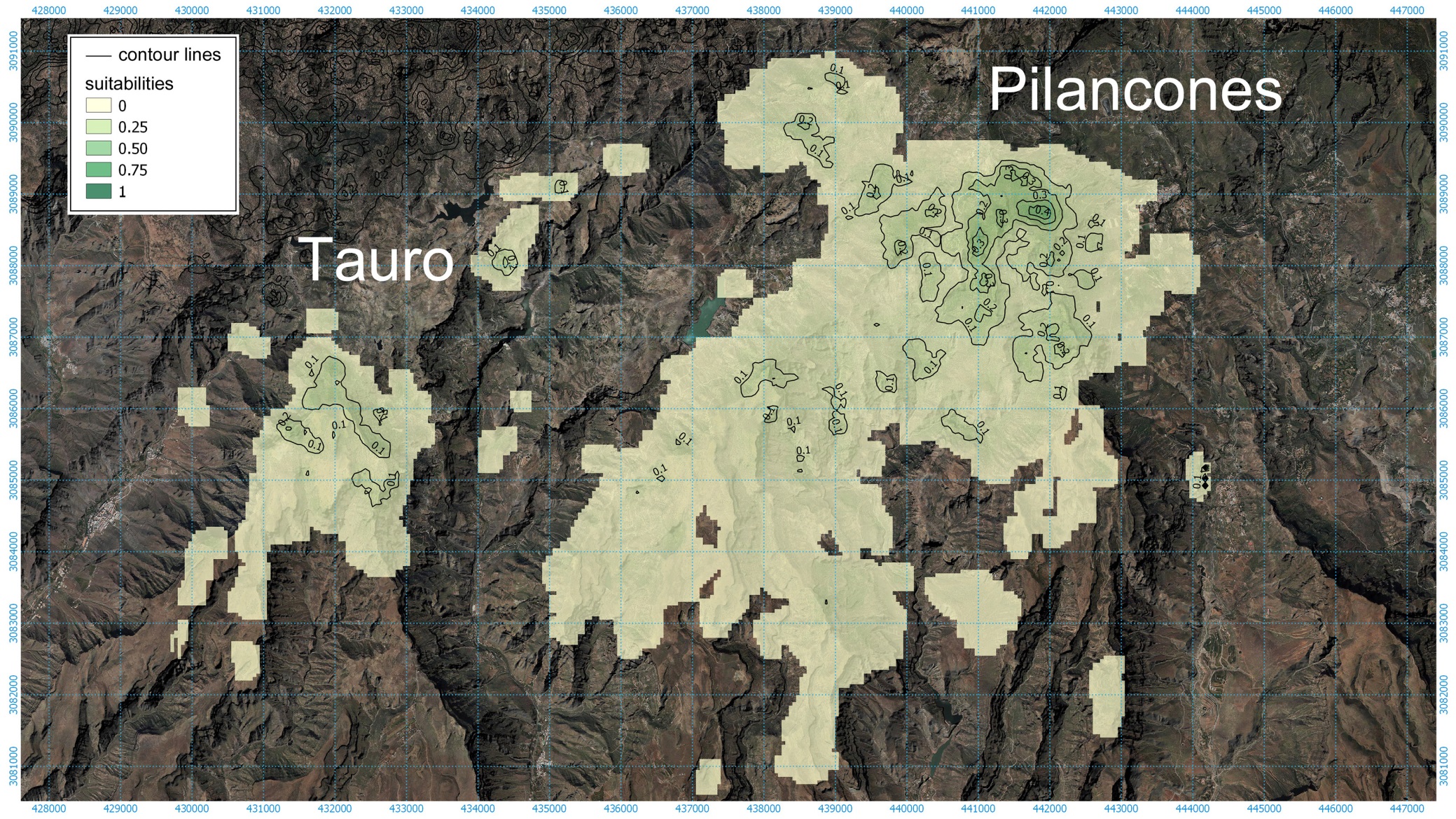

Supplement: Supplemental Information 1 — Tables S1 and Figures S1, S2 and S3. [file peerj-05-3771-s001.doc]
